# Characteristics of maxillofacial injuries resulting from road traffic accidents – a 5 year review of the case records from Department of Maxillofacial Surgery in Katowice, Poland

**DOI:** 10.1186/1746-160X-2-27

**Published:** 2006-08-28

**Authors:** Piotr Malara, Beata Malara, Jan Drugacz

**Affiliations:** 1 Department of Maxillofacial Surgery, Silesian Medical Academy, 20/24 Francuska Street, 40-027 Katowice, Poland; 2 Department of Environmental Medicine and Epidemiology, Silesian Medical Academy, 19 Jordana Street, 41-808 Zabrze, Poland

## Abstract

**Background:**

In spite of employing numerous devices improving the safety in motor vehicles, traffic accidents are still among the main reasons of maxillofacial injuries. The maxillofacial injuries remain the serious clinical problem because of the specificity of this anatomical region. The knowledge of etiologic factors and mechanisms of injuries can be helpful in a satisfactory trauma prevention. The aim of this study was to find out the incidence and the pattern of maxillofacial injuries resulting from traffic accidents in the patients treated in the Department of Maxillofacial Surgery (Silesian Medical Academy in Katowice, Poland) from January 2001 to December 2005.

**Methods:**

The material consisted of 1024 case records of patients with maxillofacial injuries treated in the Maxillofacial Surgery Department of Silesian Medical Academy. The detailed analysis was carried out on the case records of 198 patients in the age of 3 to 68 with maxillofacial injuries resulting from traffic accidents. On the basis of data from a history, examination on admission, consultations and radiological examinations, patients' age and gender, we obtained the information on a pattern of injury and detailed description of an accident (the date and the time of an accident, the role of the patient in an accident).

**Results:**

The traffic accidents were the cause of 19,93% maxillofacial injuries in the analyzed period of time. Most of the patients had injuries to the soft tissues of the face (22,21%), followed by tooth and alveolar process injuries (20,71%) and mandibular fractures (18,69%). All the types of injuries were more common in men than in women. The majority of the patients were car drivers followed by car passengers, pedestrians, cyclists and motor cyclists. The peak age of the patients was between 18 to 25 years. The prevalent number of accidents resulting in injuries to this region took place in spring, especially between noon and 4 PM.

**Conclusion:**

Our results exhibit that road traffic accidents remain among the main reasons of maxillofacial injuries following the traumas resulting from assaults and interpersonal violence. This succession of etiologic factors is in accordance with the data from the most developed countries. The relatively high incidence of injuries resulting from traffic accidents indicates the necessity to reinforce legislation aimed to prevent road traffic crashes and thus to reduce maxillofacial injuries among children and adults.

## Background

Traffic accidents are among the main etiologic factors of maxillofacial injuries and according to the results of previous research works they are the reasons of 34,42% to 90,15% of all the skeletal and soft tissues injuries of the face [1-3]. The other significant etiologic factors of injuries to the maxillofacial region are assaults [2] and sport injuries [4]. The etiology of maxillofacial injuries varies from one country to another and even within the same country depending on the prevailing socioeconomic, cultural and environmental factors. Periodic verification of the etiology of maxillofacial injuries helps to recommend ways in which maxillofacial injuries can be averted [5].

The maxillofacial injuries remain the serious clinical problems because of the specificity of this anatomical region. This is the area where the important organs are located and where the digestive and respiratory systems start. That is why the injuries to this region are the reasons of serious dysfunctions. Due to an anatomical proximity, together with maxillofacial injuries, the damages to the central nervous system often occur [6]. It must be emphasized that facial traumas are often the reasons of further esthetic disturbances. Thus, the psychological aspects of injuries to the maxillofacial region are of great importance [7]. That is why the special attention is focused on etiologic factors and the trauma mechanisms to successfully prevent these injuries.

One of the methods of trauma prevention among the users of motor vehicles in the most countries of the world is the obligatory fastening of seat belts. As a result of this requirement, 25% decrease in frequency of injury occurrence among the car users was observed [8]. The severity of injuries was significantly lower among the drivers and passengers who had the seat belts fastened in the moment of accident comparing to those who had not [9]. The employment of air-bag systems decreases the incidence of maxillofacial injuries in motor vehicle users subjected to traffic accidents, too. It is a fact worth emphasizing that over half of patients who suffered facial traumas, as a result of traffic accidents, were after use of alcohol or stupefacients [10].

The aim of this study is to find out the incidence and the pattern of maxillofacial injuries resulting from traffic accidents in the patients treated in the Department of Maxillofacial Surgery (Silesian Medical Academy in Katowice, Poland) from January 2001 to December 2005.

## Methods

The material consisted of 1024 case records of patients with maxillofacial injuries treated in the Maxillofacial Surgery Department of Silesian Medical Academy in Katowice, Poland, from January 2001 to December 2005. On admission the patients gave their consent to use the data obtained during the examination and treatment for further scientific projects. Katowice (density of population – 697 persons/km^2^; the number of registered cars – 46.000) [11] is the capital of Silesian province situated in the south of Poland. It is highly urbanized and industrialized region of Poland.

The detailed analysis was carried out on the case records of 198 patients with maxillofacial injuries resulting from traffic accidents. On the basis of data from a history, examination on admission, consultations and radiological examinations, patients' age and gender, we obtained information on the pattern of injury and detailed description of accident (the date and the time of accident). The data concerning the role of the patient in accident was collected.

The patients included into research (74 women and 124 men) were between 3 to 68 years of age. The authors of this article (PM and JD) were the surgeons responsible to see those patients at hospitals. The injuries which the patients suffered were grouped into soft tissue injuries, injuries of teeth and alveolar process, nasal injuries, mandibular fractures, maxillary fractures, zygomatic complex fractures, orbital "blow-out" fractures and multiple fractures of facial bone frame. Both single and multiple fractures of the mandible were included into mandibular fractures. All the fractures of maxilla according to the LeFort's classification, both unilateral and bilateral, were included into the group of maxillary fractures. The injuries which were the combination of the fractures listed above, were classified into the group of multiple fractures of facial bone frame.

According to the previous work of Wood and Freer [3], the patients were grouped into 6 age categories: from 3 to 7, from 8 to 17, from 18 to 25, from 26 to 40, from 41 to 55 and from 56 to 68 years. In case of smaller number of observations disabling the correct statistical testing, the age categories were jointed together. Concerning their role in the accident, the patients were grouped as drivers, passengers, pedestrians, cyclists and motor cyclists. The accidents were grouped into four categories depending on the season of the year and into six 4-hour periods upon the time of the day in which they had occurred.

The statistical analysis was done using Statistica v. 5.1. The statistical significance of differences in the numbers of observations (expressed proportionally) between the groups was checked by chi-square test on the p-level < 0,05.

## Results

There were 1024 patients with injuries to soft tissues and fractures of facial bone frame treated in the Maxillofacial Surgery Department of Silesian Medical Academy in Katowice, Poland, from January 2001 to December 2005, among which 198 cases (19,33%) resulted from the traffic accidents. There were 124 men (62,63%) and 61 women (37,37%) in the age of 3 to 68 years. The major cause of maxillofacial injuries were assaults and interpersonal violence (51,72%). The traffic accidents were followed by the etiologic factors such as falls (14,84%), sport injuries (8,20%) and the others.

The statistics of patients with particular types of injuries resulting from traffic accidents are listed in Table 1. The characterization of maxillary fractures according to Le Fort's classification is shown in Table 2. Most of the patients suffered from injuries to the soft tissues of the face (44 patients – 22,21%). This group was followed by tooth and alveolar process injuries (41 patients – 20,71%) and mandibular fractures (37 patients – 18,69%). Among the mandibular fractures the most frequent were multiple fractures which were observed in 24 patients (64,86% of all the mandibular fractures). In 21 patients (56,76%) there were two fracture fissures localized in premolar region and mandibular angle or condylar process on the opposite side. In 3 patients (8,10% of all the mandibular fractures) there were 3 fracture fissures localized in the body and angle of the mandible on the same side and in the condylar process on the opposite side. The other types of injuries were less frequent. It must be emphasized that all the types of injuries were more common in men than in women. In the cases of injuries to the soft tissues and mandibular fractures, the differences between the proportions of men and women were statistically significant on the p-level < 0,05.

**Table 1:** The statistics of patients with particular types of maxillofacial injuries resulting from traffic accidents.

Type of injury	General group (n = 198)	Women (n = 74)	Men (n = 124)
Injuries to the soft tissues	44 (22,21%)	13	31
Tooth and alveolar process injuries	41 (20,72%)	20	21
Mandibular fractures	37 (18,69%)	12	25
Zygomatic complex fractures	25 (12,63%)	10	15
Maxillary fractures	24 (12,12%)	9	15
Multiple fractures of facial bone frame	11 (5,55%)	4	7
Nose fractures	11 (5,55%)	4	7
Orbital "blow-out" fractures	5 (2,53%)	2	3

n – the number of patients

**Table 2:** The characterization of maxillary fractures resulting from traffic accidents.

Type of maxillary fracture	General group (n = 24)	Women (n = 9)	Men (n = 15)
Le Fort I	9	4	5
Le Fort II	6	2	4
Le Fort III	4	1	3
Le Fort I/II	3	1	2
Le Fort II/III	2	1	1

n – the number of patients

**Figure 1:**
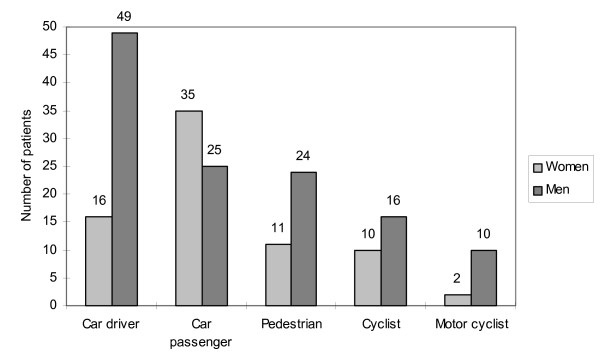
The number of patients with maxillofacial injuries resulting from traffic accidents in reference to their role in the accident.

The statistics of men and women in relation to their role in the accident are shown on Figure 1. The majority of the patients were car drivers (65 patients – 32,82%) followed by car passengers (60 patients – 30,30%), pedestrians (35 patients – 17,68%), cyclists (26 patients – 13,13%) and motor cyclists (12 patients – 6,07%). There were more women than men in the group of car passengers. In the other groups, men suffered from the injuries more often than women. In all the groups, except for the cyclists, the difference between the proportion of men and women was statistically significant on p-level < 0,05.

**Figure 2:**
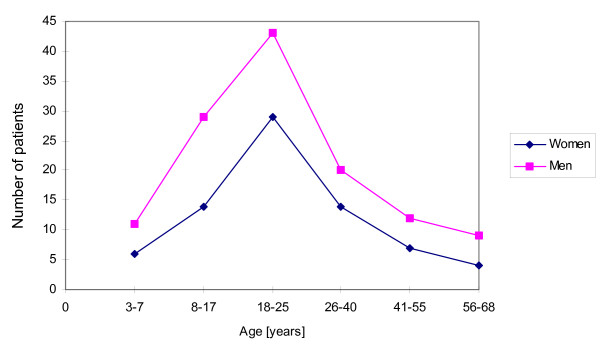
The number of patients with maxillofacial injuries resulting from traffic accidents in reference to the gender and age.

The statistics of patients with maxillofacial injuries resulting from traffic accidents in reference to their age are shown at Figure 2. Both for men and women, the injuries were the most frequent in the age of 18 to 25 (totally 72 patients – 36,36%). There were the least patients in the age of 56 to 68 (13 patients – 6,57%) and 3 to 7 (17 patients – 8,59%). In all the age groups the differences in the proportion between men and women were statistically significant on the p-level < 0,05.

**Figure 3:**
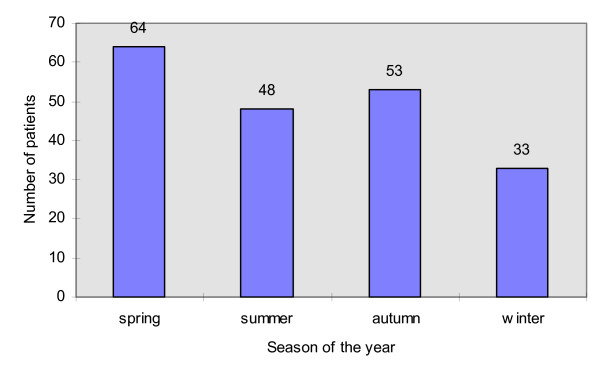
The number of patients with maxillofacial injuries resulting from traffic accidents in reference to the season of the year.

**Figure 4:**
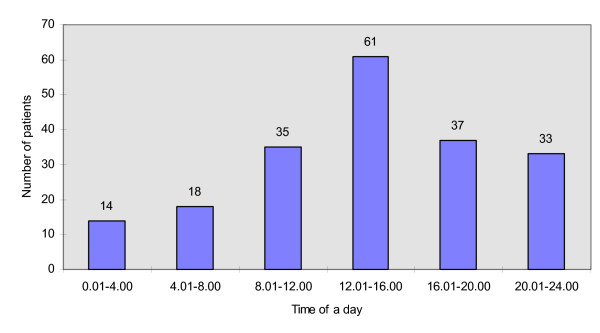
The number of patients with maxillofacial injuries resulting from traffic accidents in reference to the time of a day.

The statistics of patients with maxillofacial injuries depending on the season of the year in which the accident occurred are shown at Figure 3. This analysis revealed that the most accidents took place in spring (64 patients – 32,32%) and the least in winter (33 patients – 16,67%). There were 48 patients (24,24%) injured in summer and 53 patients (26,77%) in autumn. The analysis of the data on patients who suffered from maxillofacial injuries resulting from the traffic accidents in reference to the time of the day (shown at Figure 4) exhibited that the most of accidents occurred between midday and 4 p.m. (61 patients – 30,81%). The least patients were injured between midnight and 4 a.m. (14 patients – 7,07%).

## Discussion

Although there are many devices improving the safety of the motor vehicle users, the maxillofacial injuries resulting from traffic accidents occur quite frequently [9,12]. We found that 19,33% of maxillofacial injuries resulted from traffic accidents. It is the lower proportion comparing to those found by other authors [1-3], however, their results came from the highly developed countries with higher intensity of road traffic. We found the road traffic accidents to be the second main etiologic factor of maxillofacial injuries, following the assaults and interpersonal violence. Such a succession of etiologic factors is in accordance with the data from most developed countries [13,14]. It must be emphasized that the data from maxillofacial departments do not reflect quite precisely the incidence of maxillofacial injuries. It comes from the fact that some patients with maxillofacial injuries concomitant with severe injuries of the other body regions are admitted to neurosurgical, casualty, orthopedic, ENT and other wards [15]. Moreover, there may be no need to treat the patients with minor maxillofacial injuries in the specialized maxillofacial surgery departments.

Our results exhibit that the most common are the injuries to soft tissues followed by tooth and alveolar process injuries and mandibular fractures (Table 1). The results from Queensland (Australia) from the years 1994–1997 show that the tooth and alveolar process injuries followed by zygomatic complex fractures were the most common maxillofacial injuries resulting from traffic accidents [3]. The research covering the problem of maxillofacial injuries following steering wheel contact by drivers using seat belts exhibited that the nose fractures had the highest incidence – 43,80% [16]. Our results demonstrated much lower incidence of nose fractures (5,56%). It probably comes from the fact that most of isolated nose fractures is managed at ENT wards.

There is little information covering the problem of maxillofacial injuries in reference to the particular groups of motor vehicle users (drivers, passengers, cyclists, etc.). However, it may be verified that the men are more frequently subjected to these injuries [3]. In our research, the car passengers were the only group, in which the proportion of injured women was higher then the proportion of men (Figure 1).

Our results demonstrate that the maxillofacial injuries resulting from traffic accidents are the most common in the group of patients in the age of 18 to 25 (Figure 2). It is in accordance with other authors' findings [10,17]. Probably, it reflects inexperience and driving with dash in that age group. It is worth emphasizing that the highest incidence of maxillofacial injuries resulting from traffic accidents was noted in spring (Figure 3), when the improvement of road conditions encourages speedy driving. The lowest number of injuries was found in winter, when the collisions causing only minor injuries are more common on the roads. This result is in agreement with the research by Wood and Freer [3]. Those authors also found that the incidence of maxillofacial injuries is the highest at the afternoon rush hours, between 3 PM and 4 PM. Similarly, our data exhibit that the most of maxillofacial injuries resulting from traffic accidents occurred between noon and 4 PM, when the traffic intensity was the highest. These results support the findings of the other authors that the most of traffic accidents occur at day time, when the atmospheric conditions are relatively good [17].

## Conclusion

Our results exhibit that road traffic accidents remain among the main reasons of maxillofacial injuries following the traumas resulting from assaults and interpersonal violence. This succession of etiologic factors is in accordance with the data from the most developed countries. The most frequently occurring maxillofacial injuries resulting from traffic accidents include injuries to facial soft tissues, injuries to teeth and alveolar process and mandibular fractures. Maxillofacial injuries resulting from traffic accidents are more frequent in men than in women, especially in the age of 18 to 25 years. The highest incidence of maxillofacial injuries resulting from traffic accidents is noted in spring. The incidence of these injuries varies depending on the time of the day and is the highest between noon and 4 PM during the day rush hour. The relatively high incidence of injuries resulting from traffic accidents indicates the necessity to reinforce legislation aimed to prevent road traffic crashes and thus to reduce maxillofacial injuries among children and adults.

## Competing interests

The author(s) declare that they have no competing interests.

## Authors' contributions

PM conceived the study and did the case record search, coordinated the write-up and submission of the article. PM and JD were the surgeons responsible to see the patients at hospitals. BM carried out the literature search. PM, BM and JD participated in the writing of the manuscript. All authors have given the final approval of the version to be submitted to the journal.

## References

[B1] Haug RH, Foss J (2000). Maxillofacial injuries in the pediatric patient. Oral Surg Oral Med Oral Pathol Oral Radiol Endod.

[B2] Aksoy E, Unlu E, Sensoz O (2002). A retrospective study on epidemiology and treatment of maxillofacial fractures. J Craniofac Surg.

[B3] Wood EB, Freer TJ (2001). Incidence and aetiology of facial injuries resulting from motor vehicle accidents in Queensland for a three-year period. Aust Dent J.

[B4] Gassner R, Ulmer H, Tuli T, Emshoff R (1999). Incidence of oral and maxillofacial skiing injuries due to different injury mechanisms. J Oral Maxillofac Surg.

[B5] Adeyemo WL, Ladeinde AL, Ogunlewe MO, James O (2005). Trends and characteristics of oral and maxillofacial injuries in Nigeria: a review of the literature. Head Face Med.

[B6] Klotch DW (2000). Frontal sinus fractures: anterior skull base. Facial Plast Surg.

[B7] Hull AM, Lowe T, Finlay PM (2003). The psychological impact of maxillofacial trauma: an overview of reactions to trauma. Oral Surg Oral Med Oral Pathol Oral Radiol Endod.

[B8] Henderson MJ, Wood R (1973). Compulsory wearing of seatbelts in NSW, Australia – and evaluation of its effects on vehicle occupant deaths in the first year. Med J Aust.

[B9] Nakhgevany KH, LiBassi M, Esposito B (1994). Facial trauma in motor vehicle accidents: etiological factors. Am J Emer Med.

[B10] Hutchison IL, Magennis P, Shepherd JP, Brown AE (1998). The BAOMS United Kingdom survey of facial injuries part 1: aetiology and the association with alcohol consumption. Br J Oral Maxillofac Surg.

[B11] (2005). Silesian Province in numbers [in Polish]. Bulletin of Silesian Provincial Administration, Katowice.

[B12] Bataineh AB (1998). Etiology and incidence of maxillofacial fractures in the north of Jordan. Oral Surg Oral Med Oral Pathol Oral Radiol Endod.

[B13] King RE, Scianna JM, Petruzzelli (2004). Mandible fracture patterns: a suburban trauma center experience. Am J Otolaryngol.

[B14] Laski R, Ziccardi VB, Broder HL, Janal M (2004). Facial trauma: a recurrent disease? The potential role of disease prevention. J Oral Maxillofac Surg.

[B15] Rosman DL, Knuiman MW (1994). A comparison of hospital and police road injury data. Accid Anal Prev.

[B16] Rogers S, Hill JR, Mackay GM (1992). Maxillofacial injuries following steering wheel contact by drivers using seat belts. Br J Oral Maxillofac Surg.

[B17] Ryan GA, Legge M, Rosman D (1998). Age related changes in drivers' crash risk and crash type. Accid Anal and Prev.

